# Effect of Heat Treatment on Microstructure and Corrosion Resistance of Al-Si-Mg-Zr-Cu-Sc Alloy

**DOI:** 10.3390/ma19071422

**Published:** 2026-04-02

**Authors:** Junyi He, Jie Liu, Xiaoli Cui, Binbin Li, Xiaoqing Tian, Chao Lu, Zongshen Wang, Shan Gao, Wenqing Shi, Di Tie

**Affiliations:** 1 School of Materials Science and Engineering, Guangdong Ocean University, Yangjiang 529500, China; junyi1011@163.com (J.H.); 15803148040@163.com (J.L.); luc@gdou.edu.cn (C.L.); wangzongshen@gdou.edu.cn (Z.W.); gaoshan@gdou.edu.cn (S.G.); swqafj@163.com (W.S.); tie-di@hotmail.com (D.T.); 2 Binzhou Bohai Piston Co., Ltd., Binzhou 256602, China; twpq08@163.com; 3 School of Chemical and Energy Engineering, Binzhou Polytechnic, Binzhou 256602, China; 13326297155@163.com

**Keywords:** heat treatment, eutectic Si phase, corrosion resistance, pitting corrosion

## Abstract

Aluminum–silicon (Al-Si) alloys are widely used in aerospace, automotive manufacturing, power electronics, marine engineering and other fields due to their excellent physical properties. However, their corrosion resistance is insufficient in harsh service environments. In this study, a variety of characterization methods were adopted, including scanning electron microscopy (SEM), X-ray diffraction (XRD), electrochemical measurements (electrochemical impedance spectroscopy (EIS) and potentiodynamic polarization), immersion corrosion tests, and scanning vibrating electrode technique (SVET). The results show that the appropriate heat treatment regime can significantly enhance the corrosion resistance of the alloy, while improper aging parameters will aggravate the corrosion tendency. The optimal heat treatment regime is solution treatment at 500 °C for 4 h followed by aging at 200 °C for 48 h. Under this condition, the corrosion current density (*i_corr_*) is as low as 79.30 μA/cm^2^, and the low-frequency impedance modulus and phase angle in EIS tests are optimal. The as-extruded alloy exhibits severe localized corrosion, while the heat-treated alloy transforms into mild and uniform corrosion. The underlying mechanism is that heat treatment induces the formation of uniformly distributed nanoscale Mg_2_Si and Al_3_(Sc,Zr) precipitates, which synergistically improve the corrosion resistance of the alloy by weakening micro-galvanic coupling and facilitating the formation of a stable passive film.

## 1. Introduction

Aluminum–silicon (Al-Si) alloys are extensively utilized in aerospace, automotive manufacturing, power electronics, and marine engineering due to their favorable castability, low density, high specific strength, and good high-temperature oxidation resistance [1,2,3]. However, their broader application is constrained by insufficient corrosion resistance in harsh service environments, particularly under conditions of high humidity, in acidic media, or in marine atmospheres [4,5]. Surface degradation phenomena, including pitting corrosion, intergranular corrosion, and stress corrosion cracking, often lead to a marked reduction in service life. The corrosion behavior of Al-Si alloys is governed primarily by three interrelated factors: the alloy microstructure, the surrounding electrochemical environment, and the thermal processing history [6,7,8,9]. During corrosion, the silicon phase generally acts as a cathode, while the α-Al matrix undergoes anodic dissolution, forming micro-galvanic cells that accelerate localized attack. Heat treatment can enhance corrosion resistance by modifying the morphology and distribution of precipitates, thereby reducing the number and electrochemical activity of such micro-cells [10]. Consequently, several strategies have been proposed to improve the corrosion performance of Al-Si alloys, including the optimization of alloying elements, control of heat treatment parameters, and application of surface protection technologies [11,12,13]. In recent years, significant improvements have been achieved through the introduction of trace corrosion-resistant alloying elements combined with well-designed solution and aging treatments [14]. Nevertheless, the underlying mechanisms through which specific alloying elements and heat treatment schedules affect corrosion resistance remain incompletely understood. Further in-depth research is therefore essential to optimize alloy composition and processing routes, enabling the reliable use of high-performance Al-Si alloys in highly corrosive environments [15].

The addition of alloying elements significantly influences the corrosion resistance of Al-Si alloys [16,17,18]. Magnesium is a common strengthening addition that forms Mg_2_Si precipitates, improving mechanical properties. However, Mg_2_Si exhibits a relatively low electrode potential and is prone to localized corrosion and pitting in Cl^−^-containing media. Hence, Mg content must be optimized to balance strength with corrosion resistance. Zirconium promotes the formation of stable Al_3_Zr dispersoids, which refine grains, suppress grain boundary segregation, and effectively reduce corrosion susceptibility [19]. Moreover, Zr can influence the precipitation behavior of Cu and Mg_2_Si, weakening micro-galvanic coupling and improving pitting resistance. Copper contributes notably to solid-solution strengthening in Al-Si alloys [20], but both solute Cu and Cu-rich precipitates (e.g., Al_2_Cu) tend to act as active cathodic sites, increasing local corrosion sensitivity. Therefore, while maintaining mechanical properties, Cu content should be carefully controlled. Scandium mainly leads to the formation of nanoscale Al_3_(Sc,Zr) precipitates, which refine the grain structure, stabilize the microstructure, reduce micro-galvanic activity, and enhance overall corrosion resistance. Thus, through rational alloy design and optimization of elemental ratios, it is possible to simultaneously improve both strength and corrosion resistance. However, complex interactions among different alloying elements are likely, and further investigation is needed to clarify the specific role of precipitate formation and distribution on corrosion behavior [21].

Optimization of the heat treatment process also represents a key approach to improving the corrosion resistance of Al-Si alloys. Appropriate solution treatment promotes the dissolution of alloying elements, enhances matrix homogeneity, and reduces the amount of corrosion-sensitive phases [12]. However, excessively high solution temperatures can cause grain boundary coarsening, increasing the risk of intergranular corrosion. Aging treatments influence corrosion resistance mainly by controlling the morphology and distribution of precipitates [22]. Therefore, by integrating advanced heat treatment strategies, synergistic optimization of mechanical properties and corrosion performance can be achieved.

## 2. Materials and Methods

Al-Si-Mg-Zr-Cu-Sc alloys were prepared from Al-4Si, Al-50Mg, Al-10Zr, Al-50Cu, and Al-2Sc master alloys. The preparation procedure was as follows: First, Al-4Si master alloy was melted in a resistance furnace at 780 ± 10 °C. Other master alloys were then added to the molten aluminum, and the melt was stirred with graphite rods to remove slag and degas. After holding for 30 min, the melt was cast into an ingot with a diameter of 85 mm. The ingot was homogenized at 420 °C for 6 h in a resistance furnace, followed by hot extrusion into a rod of 9.5 mm diameter. The chemical composition of the prepared alloy is listed in Table 1.

In this study, 180 °C, 200 °C and 220 °C were selected as the aging temperatures with 0, 6, 12, 24 and 48 h as the aging durations, and the selection of this temperature range was determined based on the heat treatment characteristics of Al-Si-Mg series alloys, the diffusion law of alloying elements and the results of preliminary pre-experiments. This temperature range is not only the optimal aging window for the classic T6 heat treatment of Al-Si-Mg series alloys, which can balance the nucleation and growth processes of the Mg_2_Si strengthening precipitates, but also falls within the diffusion kinetic window (170~230 °C) of Sc and Zr atoms in the Al matrix, which can effectively promote the uniform nucleation and dispersed distribution of Al_3_(Sc,Zr) nano-precipitates. The results of preliminary experiments further verified that when the aging temperature is lower than 180 °C, the thermal motion capacity of atoms inside the alloy is insufficient and the slow diffusion of solute atoms will lead to inadequate precipitation of precipitates, while a temperature higher than 220 °C is prone to induce the coarsening of nucleated precipitates, and also cause the segregation of some solute atoms at grain boundaries and aggravate the micro-galvanic corrosion tendency of the alloy. Therefore, 180 °C, 200 °C and 220 °C were finally determined as the aging temperature variables in this study. The aging treatment parameters of this study are summarized in Table 2.

Specimens for microstructure analysis and corrosion testing were prepared by sectioning 10 mm long rod samples via wire electrical discharge machining (EDM, DK7740, Changde Machinery Manufacturing Co., Ltd., Changde, China). The samples were then cold-mounted, sequentially ground using 600, 1000, 1500, 2000, and 3000 grit sandpaper, ultrasonically cleaned (JP-020, Shenzhen Jieqing Cleaning Equipment Co., Ltd., Shenzhen, China), rinsed with anhydrous ethanol, and finally air-dried. Microstructure and elemental distribution were examined using a high-resolution field-emission scanning electron microscope (FE-SEM, Apreo 2S, Thermo Fisher Scientific, Waltham, MA, USA) equipped with an energy-dispersive X-ray spectroscopy detector (EDS, EDAX). Phase composition before and after corrosion was characterized by X-ray diffraction (XRD, XRD-7000, Shimadzu, Kyoto, Japan) performed at a scanning speed of 5°/min over a 2θ range of 10–90°.

Electrochemical investigations, consisting of electrochemical impedance spectroscopy (*EIS*) and potentiodynamic polarization measurements, were performed using a CHI660E electrochemical workstation with a conventional three-electrode configuration. The electrolyte was a 3.5 wt% NaCl solution (pH 6.8–7.2) maintained at 25 °C. The working electrode was a mounted alloy sample with an exposed surface area of 1 cm^2^, the counter electrode was a platinum sheet (10 mm × 10 mm × 0.2 mm), and the reference electrode was a saturated calomel electrode (*SCE*). The electrolyte volume-to-sample area ratio exceeded 100 mL/cm^2^. Prior to each test, the sample was immersed in the solution for 30 min to attain a stable open-circuit potential (*OCP*). Following *OCP* stabilization, potentiodynamic polarization was conducted by scanning the potential from −160 mV to +160 mV (vs. *OCP*) at a rate of 1.0 mV/s to record the polarization curve.

The corrosion potential and corrosion current density were determined using the Tafel extrapolation method. Electrochemical impedance spectroscopy (*EIS*) measurements were conducted at the open-circuit potential (*OCP*) by applying a sinusoidal potential signal with a frequency range from 100 kHz to 0.05 Hz and an amplitude of 5 mV RMS. The impedance spectra were acquired with 12 points collected per frequency decade. To ensure the reliability of the electrochemical data, at least two samples of each alloy were tested; results were required to be consistent between replicates, otherwise additional specimens were measured.

Immersion corrosion tests were conducted in a 3.5 wt% NaCl solution at 25 °C. Specimens with dimensions of 10 mm × 10 mm × 5 mm were prepared and immersed for periods of 5 and 10 days. Following immersion, corrosion products were removed by treating the samples for 3 min at 25 °C in a solution consisting of 70 vol.% HNO_3_ (500 mL/L) and deionized water (500 mL/L). The mass loss before and after immersion was measured using an analytical balance with an accuracy of 0.01 mg, and the corrosion rate was calculated according to Equation (1) following the ASTM G1-03 standard [23].

(1) $$C_{r}=\frac{KW}{AT\rho },$$

where *C_r_* is the corrosion rate (mm/a), *K* is a constant (6.78 × 10^4^), *W* is the mass loss (g), A is the exposed sample area (cm^2^), *T* is the immersion time (h), and *ρ* is the density of the alloy (g cm^−3^). Immersion tests were performed in triplicate for each alloy condition. The reported corrosion rate for each condition represents the average of the three measurements, with the associated error ranges (e.g., standard deviation) derived from the same replicate set. Following 10 days of immersion, the surface microstructure of the alloy samples under different heat treatment states was examined.

The extruded rods were sectioned into 10 mm diameter specimens using wire electrical discharge machining. The specimens were cold-mounted, and then sequentially ground with 400, 1200, 2000, and 3000 grit abrasive papers. Subsequently, they were ultrasonically cleaned, rinsed with anhydrous ethanol, and air-dried to prepare them for testing. Scanning Vibrating Electrode Technique (*SVET*) measurements were performed using a Princeton VersaScan micro-electrochemical system (AMETEK, Inc., Princeton, NJ, USA). The test parameters were as follows: a vibrating frequency of 80 Hz, an amplitude of 30 µm, a probe-to-surface distance of approximately 100 µm, a scanning area of 400 µm × 400 µm, and a step size of 50 µm [24,25,26].

(2) $$i=-\frac{k∆V}{∆r},$$

**Figure 1:**
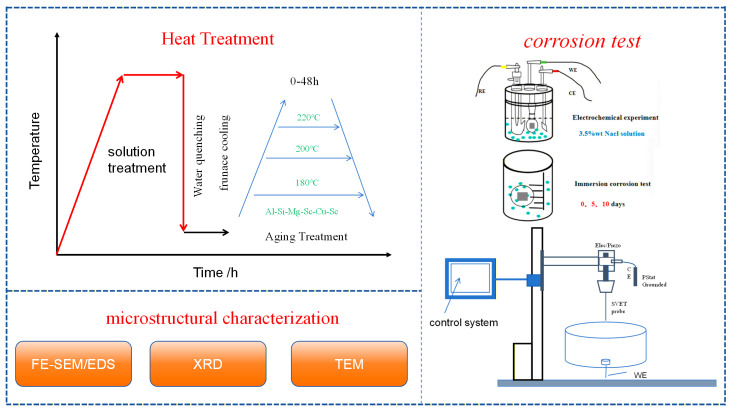
Experimental flow chart of the study.

The measured relative voltage was converted to current density using Equation (2), *i* = *k*·Δ*V*/Δ*r*, where *i* is the current density, *k* is the conductivity of the electrolyte, and Δ*V* is the potential gradient measured over a known vibration distance Δ*r*. Prior to testing, the system was calibrated to convert the local potential differences into quantitative current density values. A schematic overview of the complete experimental procedure is provided in Figure 1.

## 3. Results

### 3.1. Corrosion Behaviour

#### 3.1.1. Electrochemical Corrosion Behavior

**Figure 2:**
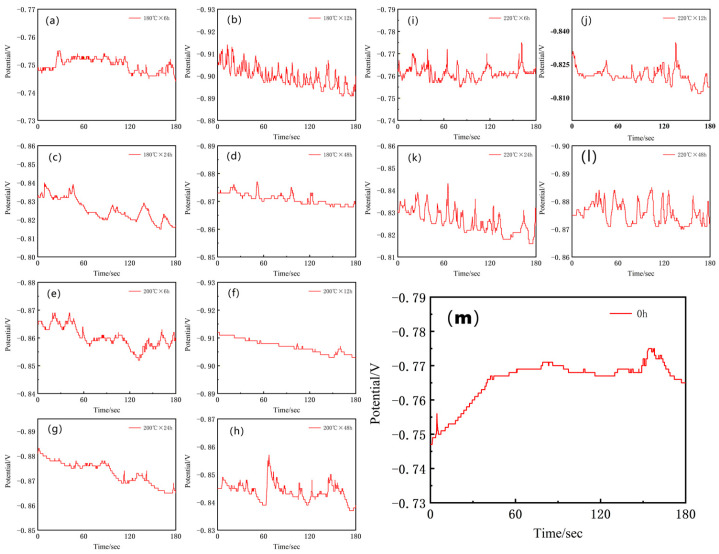
Open circuit potential (OCP) of Al-Si-Mg-Zr-Cu-Sc alloy: (**a**) 180 °C + 6 h; (**b**) 180 °C + 12 h; (**c**) 180 °C + 24 h; (**d**) 180 °C + 48 h; (**e**) 200 °C + 6 h; (**f**) 200 °C + 12 h; (**g**) 200 °C + 24 h; (**h**) 200 °C + 48 h; (**i**) 220 °C + 6 h; (**j**) 220 °C + 12 h; (**k**) 220 °C + 24 h; (**l**) 220 °C + 48 h; (**m**) 0 h.

Following the microstructural and compositional characterization, electrochemical corrosion tests were performed to evaluate the corrosion performance of the alloy. Prior to the potentiodynamic polarization and electrochemical impedance spectroscopy (EIS) measurements, the open circuit potential (OCP) was monitored until a stable value was achieved, which allowed the establishment of a steady-state surface condition and ensured the reliability of subsequent electrochemical data. The electrochemical OCPT data are shown in Figure 2.

**Figure 3:**
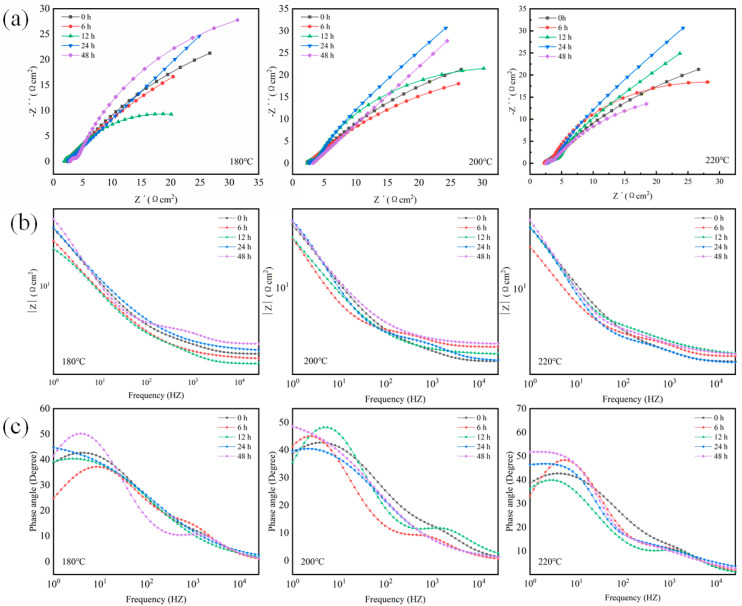
EIS results of Al-Si-Mg-Zr-Cu-Sc alloy: (**a**) Nyquist plots; (**b**) Bode impedance plots; (**c**) Bode phase angle plots.

To explore the effect of aging treatment on the corrosion resistance of the two alloys, electrochemical impedance spectroscopy (*EIS*) and potentiodynamic polarization tests were performed on samples subjected to diverse aging temperatures and durations. The EIS results are illustrated in Figure 3, presented as Nyquist plots, bode magnitude plots, and Bode phase angle plots.

Figure 3a depicts the Nyquist plots of the Al-Si-Mg-Zr-Cu-Sc alloy. For the sample aged at 180 °C, the curve exhibits a small inductive loop in the low-frequency region, followed by a large capacitive semicircle. This inductive loop originates from the relaxation effects during anodic dissolution and the adsorption of corrosion products, which induce localized active dissolution of the oxide film at surface defects. As observed from the figure, the inductive loop gradually fades with increasing aging temperature and nearly vanishes at 220 °C. This phenomenon highlights the significant influence of aging temperature on the corrosion mechanism, as higher aging temperatures facilitate the formation of a more stable surface oxide film and promote charge transfer processes across the interface.

The capacitive semicircle corresponds to the charge-transfer resistance on the alloy surface, with a larger diameter indicating a higher charge-transfer resistance and better corrosion resistance, which is highly consistent with the *Rp* values in Table 3. The variation trend of the capacitive semicircle diameter differs significantly at different aging temperatures: (1) For samples aged at 200 °C, the semicircle diameter decreases first at 6 h, and then increases continuously with prolonged aging time, reaching the maximum at 48 h. (2) For samples aged at 180 °C, the semicircle diameter decreases at 6 h, rises to the maximum at 24 h, and then decreases significantly at 48 h. (3) For samples aged at 220 °C, the semicircle diameter reaches the maximum at 6 h, plummets at 12 h, and then fluctuates with extended aging time.

The Bode magnitude plots of the Al-Si-Mg-Zr-Cu-Sc alloy are presented in Figure 3b. For aluminum alloy corrosion systems, the low-frequency impedance modulus |Z| is a core indicator for evaluating the overall corrosion resistance of the material, whose variation trend is highly consistent with the polarization resistance (*Rp*) and corrosion current density (*i_corr_*) obtained from the preceding potentiodynamic polarization test (Table 3). Specifically, a higher |Z| value in the low-frequency region corresponds to a larger *Rp*, lower *i_corr_*, slower corrosion kinetics, and thus superior corrosion resistance of the alloy [27]. For samples aged at 200 °C, the low-frequency |Z| value increases continuously with prolonged aging duration and peaks at 48 h, which is fully consistent with the trend of *Rp* rising from 56.9 Ω·cm^2^ (6 h) to 484.2 Ω·cm^2^ (48 h) and icorr decreasing from 598.9 μA/cm^2^ (6 h) to 79.30 μA/cm^2^ (48 h) at the same temperature, confirming the optimal corrosion resistance under this condition. For samples aged at 180 °C, the low-frequency |Z| value decreases at 6 h, rises to the peak at 24 h, and then drops significantly at 48 h, which is in good agreement with the evolution that *Rp* reaches the maximum of 505.0 Ω·cm^2^ and icorr drops to the minimum of 79.84 μA/cm^2^ at 24 h in Table 3, accurately reflecting the non-monotonic change in corrosion resistance with aging time at this temperature. For samples aged at 220 °C, the low-frequency |Z| value peaks at 6 h of aging, plummets at 12 h, and then fluctuates with extended aging time, which is highly consistent with the sharp drop of *Rp* from 416.3 Ω·cm^2^ (6 h) to 104.1 Ω·cm^2^ (12 h) and the surge of icorr from 98.36 μA/cm^2^ (6 h) to 502.1 μA/cm^2^ (12 h), clearly revealing the adverse effect of improper aging parameters on corrosion resistance. The above variation rule of low-frequency |Z| is also highly consistent with the change in capacitive semicircle diameter in the Nyquist plots (Figure 3a), realizing mutual verification of the two core electrochemical test results. In addition, the impedance in the high-frequency region is closely related to the compactness and stability of the surface oxide film on the alloy; among all test conditions, the sample aged at 200 °C for 48 h exhibits the highest high-frequency |Z| value, further confirming that this optimal heat treatment regime enables the formation of a more compact and protective passive film on the alloy surface.

Figure 3c presents the Bode phase angle plots of the Al-Si-Mg-Zr-Cu-Sc alloy. The evolution of the phase angle reflects systematic changes in the interfacial electrochemical properties. Generally, a higher phase angle, especially in the low-to-medium frequency region, indicates stronger interfacial capacitive characteristics that are closely related to improved corrosion resistance. A phase angle approaching −90° represents a nearly ideal capacitive response, typically corresponding to a passive film with excellent protective performance. The phase angle variation trends are consistent under the three aging temperatures. The phase angle of the as-extruded unaged sample in the low-frequency region is approximately −40°, and the sample aged at 200 °C for 48 h shows the highest phase angle among all test conditions, reaching nearly −50° in the low-to-medium frequency region. This indicates that the optimal heat treatment regime significantly enhances the capacitive characteristics of the alloy-electrolyte interface, corresponding to the formation of a compact and protective passive film.

**Figure 4:**
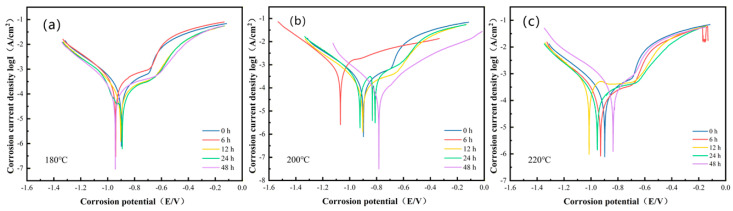
Potentiodynamic polarization curves of the Al-Si-Mg-Zr-Cu-Sc alloy under different aging conditions: (**a**) 180 °C, (**b**) 200 °C, (**c**) 220 °C.

Potentiodynamic polarization tests were conducted on the Al-Si-Mg-Zr-Cu-Sc alloy subjected to various aging temperatures and durations, with the resulting curves shown in Figure 4. These curves indicate that the corrosion potential *E_corr_* of the alloy under different heat-treatment conditions lies within the range of approximately −0.8 to −1.0 V (vs. SCE). Both *E_corr_* and corrosion current density *i_corr_* reflect the influence of aging parameters on corrosion resistance: a more positive *E_corr_* indicates a lower thermodynamic tendency of corrosion, while a lower *i_corr_* corresponds to slower corrosion kinetics, both of which are consistent with the variation trend of low-frequency |Z| in EIS tests.

At aging temperatures of 200 °C, *E_corr_* shifted toward more noble (more positive) values with prolonged aging time. After 48 h of aging, the most positive *E_corr_* and the lowest *i_corr_* were obtained, demonstrating optimal corrosion resistance under these conditions, which is in good agreement with the highest low-frequency |Z| and phase angle values obtained from EIS measurements. For example, the *i_corr_* of the sample aged at 220 °C for 24 h is 131.6 μA/cm^2^, while the icorr values of samples aged at 200 °C and 180 °C for 48 h are 79.30 μA/cm^2^ and 127.8 μA/cm^2^, respectively.

In comparison with the as-extruded (untreated) sample, which exhibited an *i_corr_* of 177.4 μA/cm^2^, the applied aging treatments led to varied but significant changes in corrosion kinetics. Aging at 200 °C/48 h lowered *i_corr_* by approximately 55.30%, corresponding to a corrosion rate reduction of the same magnitude. Aging at 180 °C/48 h resulted in a reduction of about 27.96%. In contrast, the sample aged at 220 °C/12 h showed an increase in *i_corr_* of approximately 182.9% compared to the untreated condition. This indicates that, while prolonged aging at moderate temperatures (180–200 °C) substantially enhances corrosion resistance, the specific regime of 220 °C/12 h may promote adverse microstructural features that inadvertently accelerate corrosion kinetics.

For the samples aged at 180 °C, the corrosion resistance shows a non-monotonic change with aging time, which is attributed to the staged precipitation behavior of the second phases at this temperature. The optimal corrosion resistance is achieved at 24 h of aging, while prolonged aging to 48 h leads to the deterioration of corrosion resistance, which is consistent with the variation trend of EIS results.

#### 3.1.2. Immersion Corrosion

**Figure 5:**
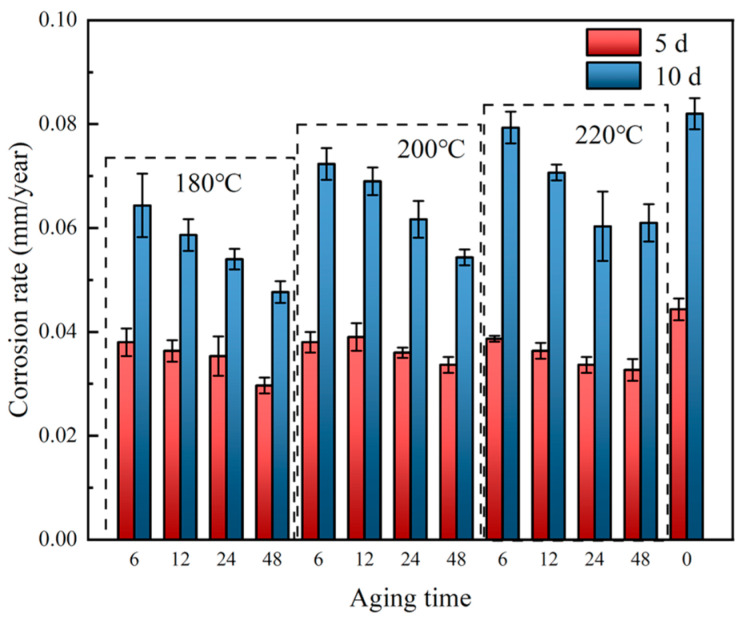
Corrosion rate of Al-Si-Mg-Zr-Cu-Sc alloy after soaking in 3.5 wt% NaCl solution for 5 and 10 days.

The corrosion rate of the alloy shows a significant non-monotonic change with aging temperature and aging time, as shown in Figure 5. For the samples aged at 180 °C, the corrosion rate decreases first and then increases with the extension of aging time, reaching the lowest value at 24 h of aging. For the samples aged at 200 °C, the corrosion rate decreases continuously with prolonged aging time, and the lowest corrosion rate is achieved at 48 h, which corresponds to the optimal corrosion resistance. For the samples aged at 220 °C, the corrosion rate increases significantly after 12 h of aging, showing an adverse effect on corrosion resistance.

### 3.2. Microstructure Analysis

#### Nanoscale Precipitate Characterization

To clarify the micro-mechanism of heat treatment regulating the corrosion resistance of the Al-Si-Mg-Zr-Cu-Sc alloy, TEM characterization was performed on the sample under the optimal heat treatment regime (500 °C for 4 h + 200 °C for 48 h).

**Figure 6:**
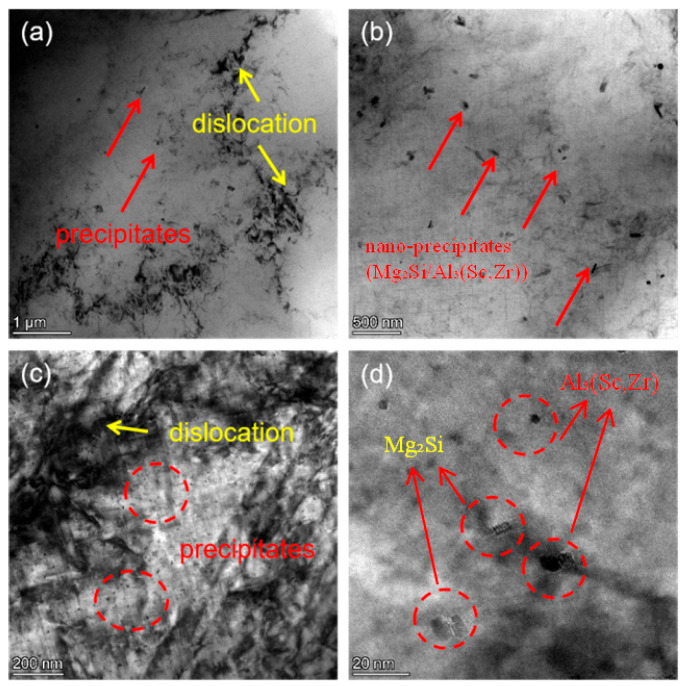
TEM microstructure of the alloy in the optimal heat treatment state (500 °C/4 h + 200 °C/48 h) (**a**,**b**) Morphology of dislocations and precipitates at low magnification; (**c**,**d**) Morphology of dislocations and precipitates at high magnification.

As shown in Figure 6a,b, a large number of nanoscale precipitates are uniformly dispersed in the α-Al matrix, with no obvious segregation at grain boundaries. The precipitates are mostly fine spherical or short rod-like particles with a size of less than 20 nm (Figure 6c,d). Combined with the alloy composition and XRD results, these uniformly distributed nano-precipitates are mainly composed of Mg_2_Si strengthening phase and Al_3_(Sc,Zr) composite phase.

The uniform dispersion of nano-precipitates is the core microstructural basis for the improved corrosion resistance of the alloy. On the one hand, the uniform precipitation of solute elements (Mg, Si, Sc, Zr) eliminates the segregation of alloying elements in the matrix, reduces the electrochemical inhomogeneity of the α-Al matrix, and weakens the micro-galvanic coupling effect between the second phase and the matrix. On the other hand, the fine and dispersed precipitates avoid the formation of large-size cathode/anode phases, which can inhibit the initiation and propagation of localized pitting corrosion, and facilitate the formation of a continuous and stable passive film on the alloy surface.

**Figure 7:**
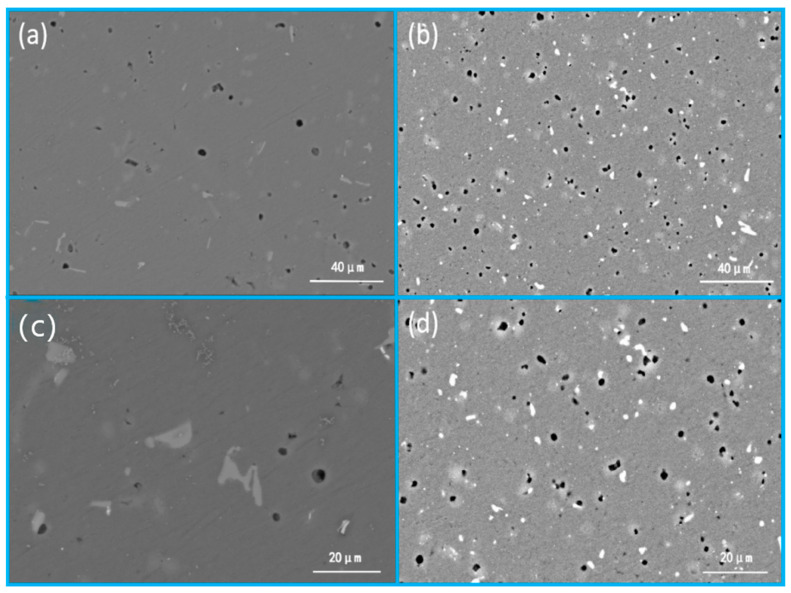
Microstructure of the alloy in the as-extruded state and optimal heat treatment state (500 °C/4 h + 200 °C/48 h) (**a**,**c**) as extruded (**b**,**d**) heat treatment.

Figure 7 characterizes the microstructural evolution of the Al-Si-Mg-Zr-Cu-Sc alloy before and after the optimal heat treatment (500 °C for 4 h + 200 °C for 48 h), with Figure 7a,c showing the as-extruded initial microstructure, and Figure 7b,d presenting the optimized microstructure after heat treatment. In the as-extruded state displayed in Figure 7a,c, the α-Al matrix contains coarse, irregular eutectic Si particles and large-sized second phases with extremely poor dispersion uniformity, which act as strong cathodic sites relative to the surrounding aluminum matrix, intensify the micro-galvanic coupling effect, and provide abundant preferential initiation sites for pitting corrosion, thus fundamentally increasing the corrosion susceptibility of the alloy. After the optimized solution-aging treatment, the microstructure in Figure 7b,d achieves significant homogenization and refinement: the size of eutectic Si and second-phase particles is markedly reduced, their distribution in the matrix becomes more uniform, and fine secondary phases are homogeneously precipitated throughout the matrix. This refined and uniform microstructure effectively weakens the micro-galvanic effect between the second phase and the matrix, inhibits the initiation and propagation of pitting corrosion, and thus achieves a substantial improvement in the corrosion resistance of the alloy.

**Figure 8:**
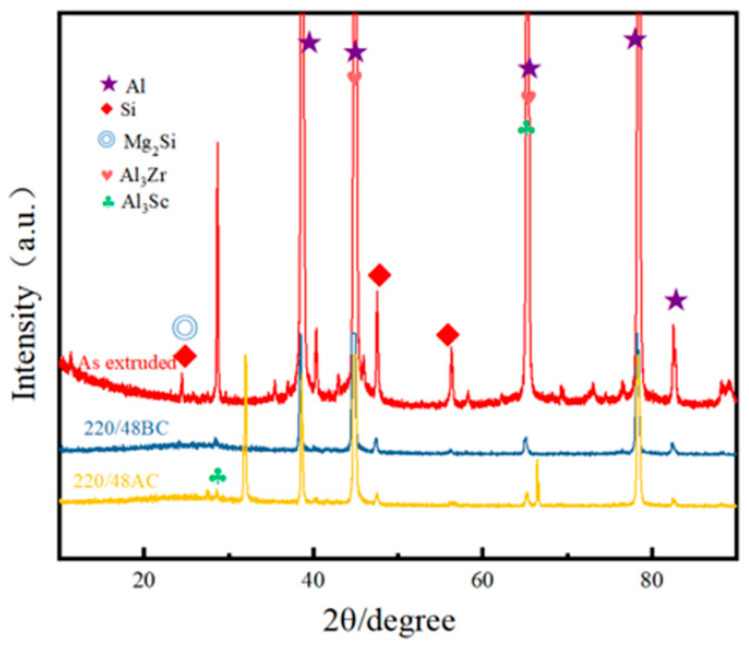
XRD patterns of the Al-Si-Mg-Zr-Cu-Sc alloy before and after immersion corrosion.

In order to further analyze the corrosion behavior of Al-Si-Mg-Zr-Cu-Sc alloy after different heat treatments, the phase composition, microstructure evolution, and element distribution characteristics of the alloy before and after immersion experiments under different heat treatment states were analyzed. Figure 8 shows the XRD of the alloy used in the immersion experiment. From Figure 8, it can be seen that before immersion corrosion, the peaks in the Al-Si-Mg-Zr-Cu-Sc alloy are mainly the peaks of Al and Si phases, with the rest being diffraction peaks of strengthening phases such as Mg_2_Si, Al_3_Zr, and Al_3_Sc. Before immersion corrosion, the diffraction peaks of the alloy are mainly from Al matrix and Si phase, as well as the diffraction peaks of strengthening phases such as Mg_2_Si, Al_3_Zr and Al_3_Sc. After immersion corrosion, the diffraction peak intensity of Mg_2_Si phase decreases significantly. This is because Mg_2_Si phase has a lower electrode potential than the α-Al matrix, which acts as the anodic phase during corrosion and undergoes preferential dissolution in the Cl^−^-containing environment. While the peak intensity of Al_3_Zr and Al_3_Sc phases has no obvious change, as these phases are noble cathode phases with high electrode potential and will not undergo preferential dissolution during the corrosion process. No obvious diffraction peaks of new corrosion products are detected, which may be due to the low content or poor crystallinity of the corrosion products.

**Figure 9:**
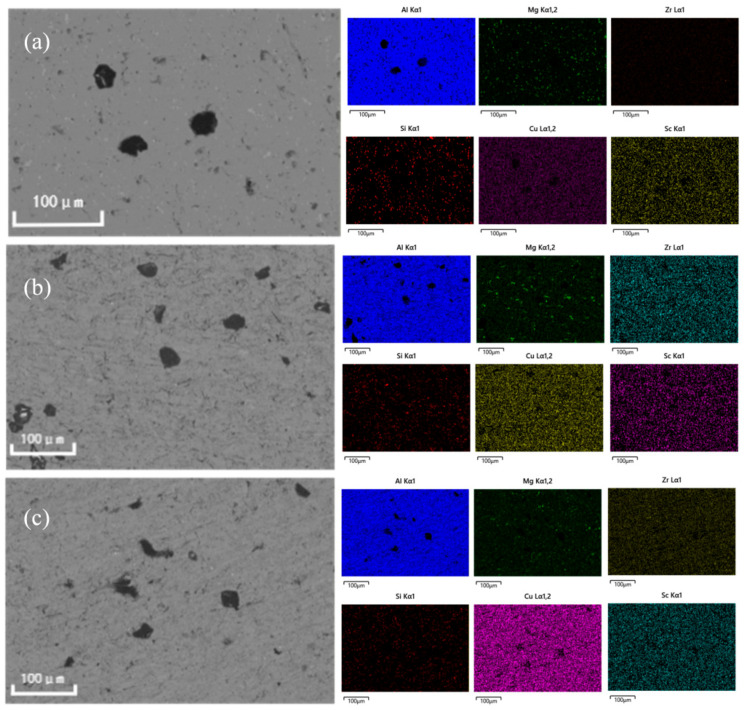
Surface corrosion morphologies of the Al-Si-Mg-Zr-Cu-Sc alloys after 10 days of immersion in the 3.5 wt% NaCl solution, (**a**) as-extruded, (**b**,**c**) 500 °C/4 h + 200 °C/48 h.

A further analysis of the microstructural evolution and elemental distribution was conducted on alloys in different heat-treatment states before and after corrosion. Figure 9 presents the surface corrosion morphology of the as-extruded alloy and the Al-Si-Mg-Zr-Cu-Sc alloy subjected to a T6 treatment (solution at 500 °C/4 h + aging at 200 °C/48 h) after 10 days of immersion in 3.5 wt% NaCl solution. Severe corrosion is evident in the as-extruded alloy. This can be attributed to the inhomogeneous distribution of coarse second phases and the segregation of solute atoms in the matrix, which aggravate the micro-galvanic coupling effect and localized intergranular attack at grain boundaries, which exacerbates localized intergranular attack. In contrast, the T6-treated alloy exhibits a more uniform corrosion morphology. The heat treatment promotes the uniform precipitation of solute elements, reducing their concentration in the matrix and minimizing micro-galvanic coupling. This results in a lower tendency for localized corrosion and improved overall corrosion resistance. Furthermore, the homogeneous distribution of precipitates mitigates the risk of preferential grain-boundary attack. The formation of a surface layer primarily composed of Al_2_O_3_ and other oxides after heat treatment is also indicated by the increased oxygen content shown in Figure 9c.

**Figure 10:**
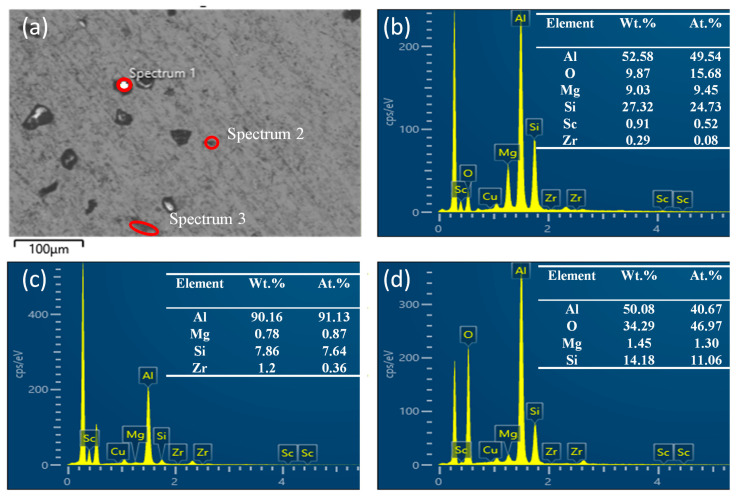
Corrosion morphology of Al-Si-Mg-Zr-Cu-Sc alloy after aging at 200 °C/48 h and soaking for 5 days. (**a**) SEM secondary electron image showing analyzed micro-regions, (**b**) Spectrum 1 (**c**) Spectrum 2 (**d**) Spectrum 3.

Further observation of corrosion morphology was conducted on the alloy after immersion. Figure 10 shows the microstructure of the Al-Si-Mg-Zr-Cu-Sc alloy following corrosion, revealing corrosion pits of various shapes and sizes on the surface. This pitting is attributed to the attack of Cl^−^ ions on the matrix adjacent to second-phase particles, which establishes local anodic sites and initiates pitting during immersion. Composition analysis of the corroded regions was conducted via EDS. Spectrum 1 (Area 1) is mainly composed of Al, Si, Mg and O elements, which corresponds to the corrosion product layer formed after the dissolution of Mg_2_Si phase and adjacent α-Al matrix, rather than the original Mg_2_Si phase. Spectrum 2 (Area 2) is the corroded α-Al matrix with a small amount of residual Si phase. Spectrum 3 (Area 3) has a high oxygen content, which corresponds to the Al_2_O_3_-rich passive film/corrosion product layer formed on the alloy surface after corrosion.

### 3.3. Scanning Vibrating Electrode Technique

**Figure 11:**
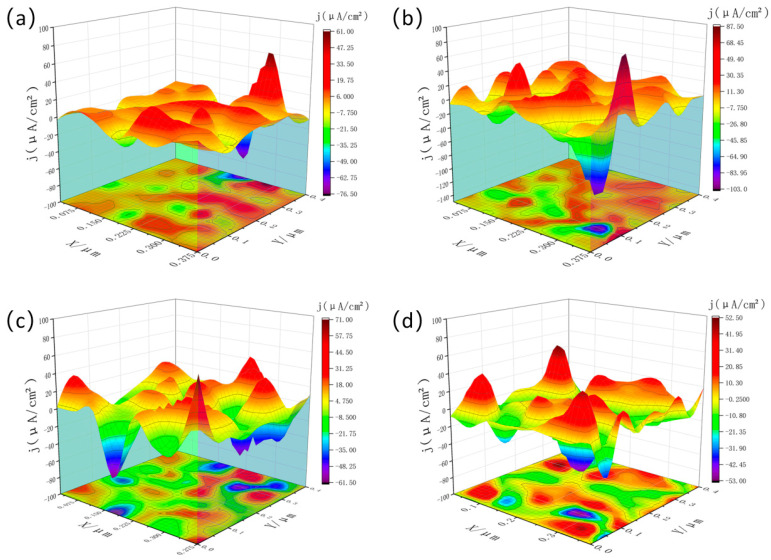
SVET map of the before-heat-treated sample (**a**) 30 min (**b**) 12 h (**c**) 18 h (**d**) 24 h.

**Figure 12:**
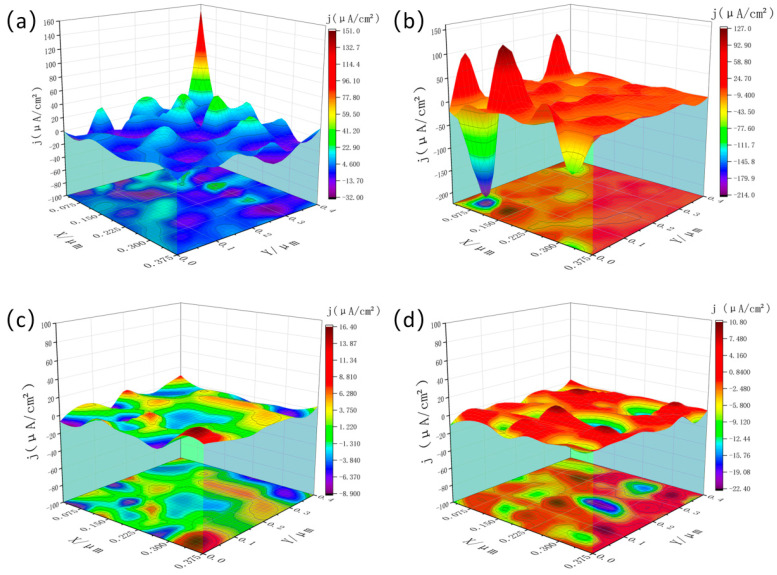
SVET map of the after-heat-treated sample (**a**) 30 min (**b**) 12 h (**c**) 18 h (**d**) 24 h.

Figure 11 and Figure 12 present the three-dimensional SVET current-density maps of the Al-4Si-1.1Mg-0.13Zr-0.06Cu-0.15Sc alloy in the as-extruded state and after the optimal T6 treatment (500 °C/4 h + 200 °C/48 h), respectively, following immersion in 3.5 wt% NaCl for 0.5, 12, 18, and 24 h.

In the as-extruded condition, the surface showed an extremely uneven current density distribution, with a large number of high-intensity anodic active sites. In contrast, the T6-treated sample showed markedly superior corrosion resistance. The as-extruded surface showed an uneven current-density distribution, with multiple high-intensity hotspots (absolute values frequently exceeding 50 µA/cm^2^ and reaching up to −106 µA/cm^2^) that persisted throughout immersion, indicating severe and sustained localized corrosion.

In contrast, the electrochemical activity of the T6-treated surface was fundamentally improved. The majority of current-density values were confined to a low range of about ±20 µA/cm^2^, and the distribution was significantly more uniform, despite isolated high-current regions. This confirms that the heat treatment effectively suppressed micro-galvanic coupling by refining and homogenizing nanoscale precipitates.

Notably, during mid-immersion (12 h), the T6-treated sample displayed isolated extreme current densities (e.g., −217 µA/cm^2^), indicating that a few local defects remained and could initiate pitting. However, these active sites did not persist; the current distribution returned to a uniformly low level after longer immersion (18 h, 24 h). This transition suggests that a more protective passive film or corrosion-product layer formed on the heat-treated surface, effectively suppressing the initially high local activity. Although the treatment may temporarily increase susceptibility to pitting, it ultimately promotes surface stabilization and “self-passivation” over time.

In summary, the SVET results demonstrate that while heat treatment does not entirely prevent pit nucleation, it substantially inhibits pit growth and propagation by creating a more homogeneous microstructure. Consequently, the corrosion mode shifts from “severe localized corrosion” in the as-extruded state to “mild and uniform corrosion” after T6 treatment—a conclusion consistent with the improvements observed in EIS and potentiodynamic polarization data.

## 4. Discussion

This study systematically investigated the influence of heat treatment on the microstructure and corrosion resistance of an Al-Si-Mg-Zr-Cu-Sc alloy. The experimental results demonstrated that the sample subjected to solution treatment at 500 °C for 4 h followed by aging at 200 °C for 48 h exhibited the most optimal comprehensive performance. A detailed analysis of the results under this specific condition is presented below.

**Figure 13:**
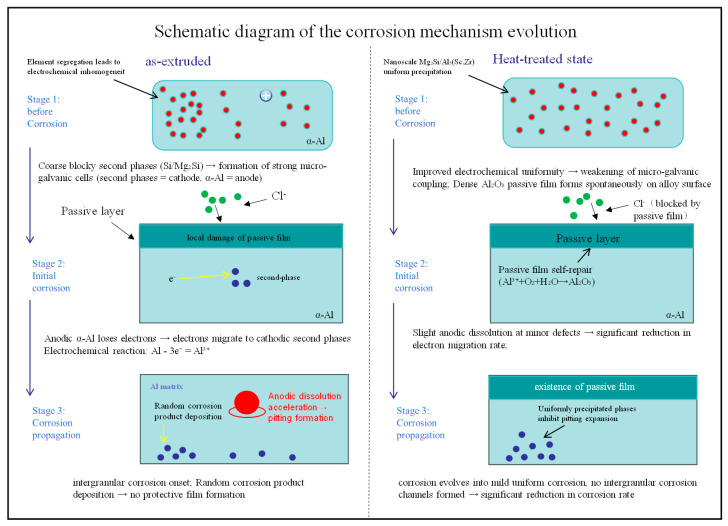
Schematic illustration of the corrosion mechanism of Al-Si-Mg-Zr-Cu-Sc alloy in as-extruded and T6-treated (500 °C/4 h + 200 °C/48 h) states in 3.5 wt% NaCl solution.

Figure 13 shows the stage-specific corrosion mechanism schematic of the as-extruded and optimally heat-treated (solution treated at 500 °C for 4 h followed by aging at 200 °C for 48 h) Al-Si-Mg-Zr-Cu-Sc alloy in a 3.5 wt% NaCl solution, which clearly illustrates the core process by which heat treatment modulates the alloy’s microstructure to achieve a fundamental transition in corrosion mode from severe localized corrosion to mild uniform corrosion. The corrosion behaviors and corresponding microstructural regulation mechanisms at each stage are elaborated as follows:

(1) Pre-corrosion Stage: Electrochemical intrinsic properties dictated by the microstructure

In the as-extruded alloy, coarse and inhomogeneously distributed eutectic Si phases and large-sized second-phase particles generate intense electrochemical heterogeneity with the α-Al matrix, and such inhomogeneity is further exacerbated by solute segregation at grain boundaries. Owing to their higher electrode potential, the second-phase particles act as cathodes while the surrounding α-Al matrix serves as anodes, leading to the spontaneous formation of numerous micro-galvanic cells prior to corrosion. These cells provide the thermodynamic prerequisites for the initiation of localized corrosion (Figure 13, As-extruded, Stage 1). In contrast, for the alloy subjected to optimal heat treatment, nanoscale Mg_2_Si and Al_3_(Sc,Zr) phases precipitate uniformly within the α-Al matrix, which effectively eliminates solute segregation and second-phase coarsening, thus significantly enhancing the electrochemical uniformity of the alloy. As a result, the number and coupling strength of micro-galvanic cells are drastically reduced. Meanwhile, the homogeneous microstructure facilitates the spontaneous formation of a dense Al_2_O_3_ passive film on the alloy surface, which acts as a robust physical barrier against the infiltration of corrosive media (Figure 13, Heat-treated state, Stage 1).

(2) Corrosion Initiation Stage: Infiltration of corrosive media and initiation of electrochemical reactions

Chloride ions (Cl^−^) in the NaCl solution, as active corrosive anions, are the core inducer for the initiation of localized corrosion. The as-extruded alloy surface is unprotected by a stable passive film, allowing Cl^−^ to rapidly infiltrate the alloy matrix and react with the α-Al in the anodic regions of micro-galvanic cells. This triggers the anodic dissolution of aluminum with electron loss (Al − 3e^−^ = Al^3+^), and the released electrons migrate rapidly to the cathodic second-phase particles through the matrix. Sustained electrochemical reactions cause irreversible damage to the passive film in local areas, which subsequently serve as nucleation sites for pitting corrosion (Figure 13, As-extruded, Stage 2). For the heat-treated alloy, the dense Al_2_O_3_ passive film on its surface can effectively block the rapid infiltration of Cl^−^, resulting in only slight anodic dissolution at a small number of micro-defects. Furthermore, the excellent electrochemical uniformity of the heat-treated alloy leads to a significant reduction in electron migration rate, which largely inhibits the micro-galvanic cell reactions. Simultaneously, the dissolved Al^3+^ can react with O_2_ and H_2_O in the electrolyte to realize the self-repair of the passive film (4Al^3+^ + 3O_2_ + 6H_2_O = 2Al_2_O_3_·3H_2_O), thereby preventing the further propagation of corrosion (Figure 13, Heat-treated state, Stage 2).

(3) Corrosion Propagation Stage: Divergent evolution of corrosion modes

After the nucleation of pitting corrosion in the as-extruded alloy, the strong micro-galvanic cells formed by coarse blocky second-phase particles continuously drive the anodic dissolution of α-Al in the anodic regions, leading to the rapid expansion and deepening of pitting pits. Meanwhile, solutes segregated at grain boundaries form intergranular corrosion channels, and the synergetic effect of localized corrosion (pitting corrosion coupled with intergranular corrosion) results in a sharp increase in the corrosion rate (Figure 13, As-extruded, Stage 3). In the heat-treated alloy, the uniform distribution of nanoscale Mg_2_Si and Al_3_(Sc,Zr) phases can effectively inhibit the expansion of pitting pits, and the stable passive film persistently blocks the infiltration of corrosive media. Even if slight corrosion occurs at a small number of micro-defects, it evolves into overall uniform corrosion due to the high electrochemical uniformity of the alloy, without any obvious characteristics of localized corrosion. Ultimately, a significant reduction in the corrosion rate is achieved (Figure 13, Heat-treated state, Stage 3).

In summary, the improvement of the alloy’s corrosion performance by heat treatment is essentially attributed to the modulation of microstructural homogeneity, which weakens the micro-galvanic coupling effect from the source and simultaneously promotes the formation and self-repair of a stable protective passive film. This realizes a fundamental transition in the corrosion mechanism of the Al-Si-Mg-Zr-Cu-Sc alloy from micro-galvanic cell-driven rapid localized corrosion to passive film-modulated mild uniform corrosion.

Under the optimal heat treatment regime (500 °C for 4 h + 200 °C for 48 h), the corrosion resistance of the alloy is significantly enhanced, which is comprehensively verified by multiple test results. Electrochemical measurements (Figure 3 and Figure 4 and Table 3) show that the corrosion current density (*i_corr_*) of the optimal sample is as low as 79.30 μA/cm^2^, which is far lower than that of the sample aged at 180 °C for 48 h (127.8 μA/cm^2^). Meanwhile, the non-monotonic evolution of corrosion resistance at 180 °C—peaking at 24 h of aging and deteriorating significantly at 48 h, as evidenced by the change in capacitive semicircle diameter in Nyquist plots (Figure 3a), low-frequency |Z| value in Bode plots (Figure 3b) and *Rp*/*i_corr_* parameters in Table 3—can also be systematically clarified from the perspective of atomic diffusion kinetics and precipitation evolution, combined with microstructural characterization results.

For the aging treatment at 180 °C, the thermal driving force leads to a pronounced difference in the diffusion kinetics of distinct alloying elements in the Al matrix: Mg atoms maintain a relatively fast diffusion rate, while the migration of Sc and Zr atoms is extremely sluggish at this temperature, which directly drives the staged evolution of precipitate microstructure and corresponding corrosion resistance along with the extension of aging time. In the early stage of aging (within 24 h), Mg and Si atoms preferentially diffuse and nucleate into fine, uniformly dispersed nano-sized Mg_2_Si precipitates. This process consumes the supersaturated solute atoms in the α-Al matrix, mitigates the electrochemical inhomogeneity of the matrix, and thus weakens the micro-galvanic coupling effect. Correspondingly, the charge transfer resistance of the alloy increases continuously, reaching the maximum *Rp* of 505.0 Ω·cm^2^ and the minimum *i_corr_* of 79.84 μA/cm^2^ at 24 h (Table 3), which corresponds to the optimal corrosion resistance at this aging temperature. As the aging time prolongs to 48 h, the insufficient thermal driving force still fails to support the effective nucleation and uniform dispersion of Al_3_(Sc,Zr) precipitates—the core phase for stabilizing grain boundaries and inhibiting precipitate coarsening, as verified by the TEM characterization in Figure 6. Meanwhile, the pre-formed fine Mg_2_Si phases undergo inhomogeneous coarsening and grain boundary segregation. The coarsened second phases aggravate the micro-galvanic coupling effect with the Al matrix, and the grain boundary-segregated precipitates act as preferential corrosion channels. These two factors jointly result in a significant decrease in charge transfer resistance and the deterioration of corrosion resistance at 48 h, which is well consistent with the reduced capacitive semicircle diameter in Figure 3a and the elevated *i_corr_* in Table 3.

In contrast, 200 °C exactly falls within the optimal diffusion kinetic window of Mg, Sc and Zr atoms in the Al matrix. This temperature can not only provide sufficient driving force for the uniform nucleation and dispersed distribution of nano-scale Mg_2_Si and Al_3_(Sc,Zr) precipitates during 48 h of aging (as shown in the TEM results of Figure 6), but also avoid the obvious coarsening of the precipitates [28]. The synergistic effect of the two types of uniformly dispersed nano-precipitates minimizes the micro-galvanic coupling effect inside the alloy, and facilitates the formation of a stable and compact passive film on the alloy surface. This mechanism is further verified by the highest low-frequency |Z| value in Bode plots (Figure 3b), the uniform and mild corrosion morphology after immersion (Figure 9), and the low and evenly distributed local current density in SVET tests (Figure 12), thus achieving the optimal comprehensive corrosion resistance of the alloy. EIS measurements (as depicted in Figure 3) revealed that the heat-treated alloy exhibited higher low-frequency impedance modulus and phase angle, indicative of the formation of a stable passive film on the surface. EDS analysis confirmed an increased oxygen content on the surface of the heat-treated alloy, which was attributed to the formation of an Al_2_O_3_-based passive film. The uniform distribution of precipitates not only mitigated the micro-galvanic effect but also reduced defects within the passive film, thereby hindering the rapid penetration of Cl^−^ ions to the underlying matrix. This aligns with the research by Evertsson et al. [29,30], which concluded that a homogeneous microstructure facilitates the formation of a continuous and stable oxide film on aluminum alloys.

SVET (Scanning Vibrating Electrode Technique) was employed to observe the corrosion mechanism with higher precision. The results showed that the as-extruded alloy exhibited persistent high current density hotspots (with a peak value reaching 106 µA/cm^2^) during immersion, indicating continuous localized corrosion. In stark contrast, the current density of the heat-treated alloy was confined within the range of ±20 µA/cm^2^. Transient localized activity was observed only at 12 h of immersion, which was rapidly suppressed by the passive film. The corrosion mode thus transitioned from severe localized corrosion in the as-extruded state to relatively mild and uniform corrosion in the heat-treated state. This transformation stems from the elimination of grain boundary precipitate segregation—i.e., the corrosion-sensitive zones—through the heat treatment process.

Despite these insightful findings, this study has certain limitations. The primary focus was on the corrosion resistance of the alloy, without further investigation into its mechanical properties (such as hardness and tensile strength). Future research should aim to explore strategies that can improve the corrosion resistance of the Al-Si-Mg-Zr-Cu-Sc alloy without compromising its mechanical performance, ultimately leading to the development of an alloy with exceptional comprehensive properties.

## 5. Conclusions

The influence of heat treatment on the microstructure and corrosion resistance of an Al-Si-Mg-Zr-Cu-Sc alloy was investigated. The principal findings are summarized as follows:

1. Appropriate heat treatment can significantly enhance the corrosion resistance of the Al-Si-Mg-Zr-Cu-Sc alloy, with the optimal regime of solution treatment at 500 °C for 4 h followed by aging at 200 °C for 48 h, achieving a corrosion current density as low as 79.30 μA/cm^2^ and optimal low-frequency impedance modulus and phase angle in EIS tests.
2. The synergistic effect of combined Sc and Zr additions with heat treatment induces uniformly dispersed nanoscale Mg_2_Si and Al_3_(Sc,Zr) precipitates, which improve corrosion resistance by weakening micro-galvanic coupling and promoting the formation of a stable Al_2_O_3_ passive film.
3. Heat treatment realizes a fundamental transformation of the alloy’s corrosion mode from severe localized corrosion (peak current density up to 106 µA/cm^2^) in the as-extruded state to mild and uniform corrosion (current density within ±20 µA/cm^2^) as verified by SVET.

## Figures and Tables

**Table 1:** **Chemical** **compositions of the Al-Si-Mg-Zr-Cu-Sc alloy.**

Alloy Composition	Elements					
	Si	Mg	Zr	Cu	Sc	Al
Al-4Si-1.1Mg-0.13Zr-0.06Cu-0.15Sc	3.96	1.10	0.13	0.06	0.15	Bal

**Table 2:** **Aging** **process parameters of the Al-Si-Mg-Zr-Cu-Sc alloy.**

Alloy	Aging Temperature	Aging Time
Al-Si-Mg-Zr-Cu-Sc	180 °C, 200 °C, 220 °C	0, 6, 12, 24, 48

**Table 3:** Electrochemical parameters obtained from Tafel fitting of samples with different heat treatments in 3.5 wt% NaCl solution.

Heat Treatment Condition	*E_corr_*/mV	*β_a_*/mV·dec^−1^	*β_c_*/mV·dec^−1^	*i_corr_*/A	*j_corr_*/μA·cm^−2^	*Rp*/Ω·cm^2^
as-extruded (0 h)	−898	4.295	6.252	1.774 × 10^−4^	177.4	232.4
180 °C for 6 h	−940	4.336	6.113	2.596 × 10^−4^	259.6	160.3
180 °C for 12 h	−929	4.076	6.509	2.095 × 10^−4^	209.5	196.0
180 °C for 24 h	−892	4.535	6.250	7.984 × 10^−5^	79.84	505.0
180 °C for 48 h	−943	3.528	6.841	1.278 × 10^−4^	127.8	328.0
200 °C for 6 h	−1069	6.862	5.901	5.989 × 10^−4^	598.9	56.9
200 °C for 12 h	−903	4.190	6.828	1.028 × 10^−4^	102.8	383.8
200 °C for 24 h	−866	5.218	6.792	2.271 × 10^−4^	227.1	396.4
200 °C for 48 h	−783	4.851	6.473	7.930 × 10^−5^	79.30	484.2
220 °C for 6 h	−929	3.403	7.214	9.836 × 10^−5^	98.36	416.3
220 °C for 12 h	−1014	2.642	5.675	5.021 × 10^−4^	502.1	104.1
220 °C for 24 h	−953	3.530	6.859	1.316 × 10^−4^	131.6	318.0
220 °C for 48 h	−836	4.602	6.410	2.727 × 10^−4^	272.7	144.8

## Data Availability

The original contributions presented in this study are included in the article. Further inquiries can be directed to the corresponding author.
